# A Novel Plasmid-Encoded Serotype Conversion Mechanism through Addition of Phosphoethanolamine to the O-Antigen of *Shigella flexneri*

**DOI:** 10.1371/journal.pone.0046095

**Published:** 2012-09-26

**Authors:** Qiangzheng Sun, Yuriy A. Knirel, Ruiting Lan, Jianping Wang, Sof’ya N. Senchenkova, Dong Jin, Alexander S. Shashkov, Shengli Xia, Andrei V. Perepelov, Qiang Chen, Yan Wang, Haiyin Wang, Jianguo Xu

**Affiliations:** 1 State Key Laboratory for Infectious Disease Prevention and Control, National Institute for Communicable Disease Control and Prevention, China CDC, Changping, Beijing, China; 2 N. D. Zelinsky Institute of Organic Chemistry, Russian Academy of Sciences, Moscow, Russian Federation; 3 School of Biotechnology and Biomolecular Sciences, University of New South Wales, Sydney, Australia; 4 Branch for Enteric Disease Control and Prevention, Institute for Infectious Disease Control and Prevention, Henan Center for Disease Control and Prevention, Zhengzhou, China; Wadsworth Center, New York State Dept. Health, United States of America

## Abstract

*Shigella flexneri* is the major pathogen causing bacillary dysentery in developing countries. *S. flexneri* is divided into at least 16 serotypes based on the combination of antigenic determinants present in the O-antigen. All the serotypes (except for serotype 6) share a basic O-unit containing one N-acetyl-d-glucosamine and three l-rhamnose residues, whereas differences between the serotypes are conferred by phage-encoded glucosylation and/or O-acetylation. Serotype Xv is a newly emerged and the most prevalent serotype in China, which can agglutinate with both MASF IV-1 and 7,8 monoclonal antibodies. The factor responsible for the presence of MASF IV-1 (E1037) epitope has not yet been identified. In this study, we analyzed the LPS structure of serotype Xv strains and found that the MASF IV-1 positive phenotype depends on an O-antigen modification with a phosphoethanolamine (PEtN) group attached at position 3 of one of the rhamnose residues. A plasmid carried gene, *lpt-O* (LPS phosphoethanolamine transferase for O–antigen), mediates the addition of PEtN for serotype Xv and other MASF IV-1 positive strains. These findings reveal a novel serotype conversion mechanism in *S. flexneri* and show the necessity of further extension of the serotype classification scheme recognizing the MASF IV-1 positive strains as distinctive subtypes.

## Introduction

*Shigella flexneri* is the major pathogen causing bacillary dysentery in developing countries. It is estimated that *Shigella* is responsible for approximately 164.7 million shigellosis cases annually worldwide, resulting in 1,100,000 deaths, with the majority involving children under five years old [1].

*S. flexneri* is divided into various serotypes based on the combination of antigenic determinants present in the O-antigen of the cell envelope lipopolysaccharide (LPS). To date, at least 16 serotypes have been recognized [2], [3], [4], [5]. Except for serotype 6, all have the same basic repeating tetrasaccharide unit, comprised of →2)-α-L-Rha*p* ^III^-(1→2)-α-L-Rha*p* ^II^-(1→3)-α-L-Rha*p* ^I^-(1→3)-β-D-Glc*p*NAc-(1→ [4]. The basic O-antigen structure is referred to as serotype Y characterized by a single group 3,4 antigenic determinant. The addition of glucosyl and/or O-acetyl residues to different sugars in the tetrasaccharide unit results in the type- (i.e. I, II, III, IV, V, IC) and group- (i.e. 3,4; 6; 7,8) specific antigenic determinants [3]. The O-acetyl on Rha^I^ defines group 6 determinant in serotype 3a, 3b, 1b, and 4b strains [6], [7]. Determinants specific for type I, IC, II, IV, V and group 7,8 antigens are associated with glucosylation on various sugar residues in the tetrasaccharide unit. The genes responsible for the O-antigen glucosylation [*gtrA*, *gtrB*, and *gtr* (type)] have been identified [3], [8], [9], [10], [11]. They are arranged in a single operon known as the *gtr* cluster [12]. *gtrA* and *gtrB* are highly conserved and interchangeable, while *gtr* (type) is unique and encodes the glucosyltransferase responsible for the attaching of a glucosyl group to a specific sugar in the tetrasaccharide repeat unit of the O-antigen [3], [12]. The O-acetylation of Rha^I^ depends on the presence of the *oac* gene for O-acetyl transferase [6], [7]. All the O-antigen modification genes known to date are encoded by seven temperate bacteriophages or prophages (SfI, SfIC, SfII, Sf6, SfIV, SfV and SfX), which are integrated into the conserved sites of the host *S. flexneri* genome [3], [6], [8], [9], [10], [11], [13], [14].

In 2001, a novel *S. flexneri* serotype called serotype Xv appeared in Henan province, China. In the following years (2002–2006), it replaced serotype 2a and became the most prevalent serotype in Henan province, accounting for 14%, 35%, 47%, 48%, and 27% of the isolations in the respective years [2]. Serotype Xv was also found to be the most prevalent serotype in Shanxi province in 2006 (67%) and 2007 (33%) and in Gansu, Anhui, and Shanghai in 2007 with 67%, 54%, and 35% of the isolations of *S. flexneri*, respectively [2]. Although it has declined since 2007, serotype Xv is still the predominant serotype in China (http://www.moh.gov.cn).

Since serotype Xv can agglutinate with both group antigen-specific monoclonal antibodies MASF IV-1 and 7,8, it was initially named as serotype 4X and then as 4c [15], [16]. Compared to typical serotype X, serotype Xv strains carry an additional new O-antigenic epitope MASF IV-1 (also called E1037, an antigenic determinant specific for MASF IV-1 antibody) [2]. Such antigenic determinant has also been reported to be presented on some *S. flexneri* serotypes 4a, Y and 6 strains [15], [17]. The genes responsible for the group 7,8 antigen have been identified on a SfX prophage integrated into the host genome [2]. However, the factor(s) responsible for the presence of MASF IV-1 or E1037 antigenic determinant in serotype Xv and other MASF IV-1 positive serotypes had not yet been identified.

In this study, we analyzed the O-antigen structure of serotype Xv strains and found a phosphoethanolamine (PEtN) residue attached at position 3 of Rha^II^, which is absent from the typical serotype X O-antigen. This modification was shown to confer the MASF IV-1 positive phenotype in serotype Xv strains. The gene named as *lpt-O*, encoding an LPS phosphoethanolamine transferase for O-antigen was identified to be responsible for carrying out the PEtN modification in serotype Xv and other MASF IV-1 positive strains and was found to be carried on a plasmid.

## Materials and Methods

### Ethics Statement

This study was reviewed and approved by the ethics committee of National Institute for Communicable Disease Control and Prevention, the Chinese CDC. *S. flexneri* strains were acquired with the written informed consent of the diarrhea patients and with the approval of the ethics committee of National Institute for Communicable Disease Control and Prevention, according to the medical research regulations of Ministry of Health (permit number 2007-17-3).

### Bacterial Strains, Plasmid and Culturing Condition

Serotype Xv strains 2002017 [2] and 2003055 were used for LPS structure analysis. Serotype X strain 51580 (amp^s^) and 4a strain NCTC 9725 (amp^s^) were used as hosts for *lpt-O* gene expression. pMD20-T Vector (TaKaRa) was used for DNA sequencing and expression vector. *E. coli* JM109 was used for plasmid propagation. *S. flexneri* strains used for plasmid profiling and *lpt-O* gene detection analysis were isolated from diarrheal patients in China or purchased from National Collection of Type Cultures (NCTC), UK. Strains were grown in a 37°C incubator or orbital shaker in Luria-Bertani broth (LB) supplemented with ampicillin (100 µg ml^−1^) when appropriate.

### Isolation of Lipopolysaccharides and Polysaccharides

LPS were isolated from dried bacterial cells by the phenol-water method [18]. The crude extract without separation of the layers was dialyzed against distilled water, nucleic acids and proteins were precipitated by adding aqueous 50% trichloroacetic acid at 4°C to reduce pH to 2, the supernatant was dialyzed against distilled water and freeze-dried to give purified LPS in yields of 5.6–7.6% of dried cells mass. Delipidation of the LPS was performed with aqueous 2% acetic acid (6 ml) at 100°C until precipitation of lipid A. The precipitate was removed by centrifugation (13,000×*g*, 20 min), and the supernatant was fractionated by gel-permeation chromatography on a column (56×2.6 cm) of Sephadex G-50 Superfine (Amersham Biosciences, Sweden) in 0.05 M pyridinium acetate buffer, pH 4.5, monitored with a differential refractometer (Knauer, Germany). High-molecular mass O-polysaccharides were obtained in yields of 27–44% of LPS mass.

### NMR Spectroscopy

Samples were deuterium-exchanged by freeze-drying twice from 99.9% D_2_O and then examined as solutions in 99.95% D_2_O at 40°C. NMR spectra were recorded on an Avance II 600 spectrometer (Bruker, Germany) using internal sodium 3-(trimethylsilyl) propanoate-2,2,3,3-d_4_ (δ_H_ 0) and acetone (δ_C_ 31.45) as references. Two-dimensional NMR spectra were obtained using standard Bruker software and parameters set as described [19]. TopSpin 2.1 program was used to acquire and process the NMR data.

### Slide Agglutination Analysis

The serotypes of *S. flexneri* strains were determined by slide agglutination test using two serotyping kits specific for all *S. flexneri* type- and group-factor antigens: (i) a commercially available monovalent antisera kit (Denka Seiken, Japan) and (ii) monoclonal antibody reagents (Reagensia AB, Sweden), as described in the manufacturers’ protocols.

### DNA Techniques

**Figure 1:**
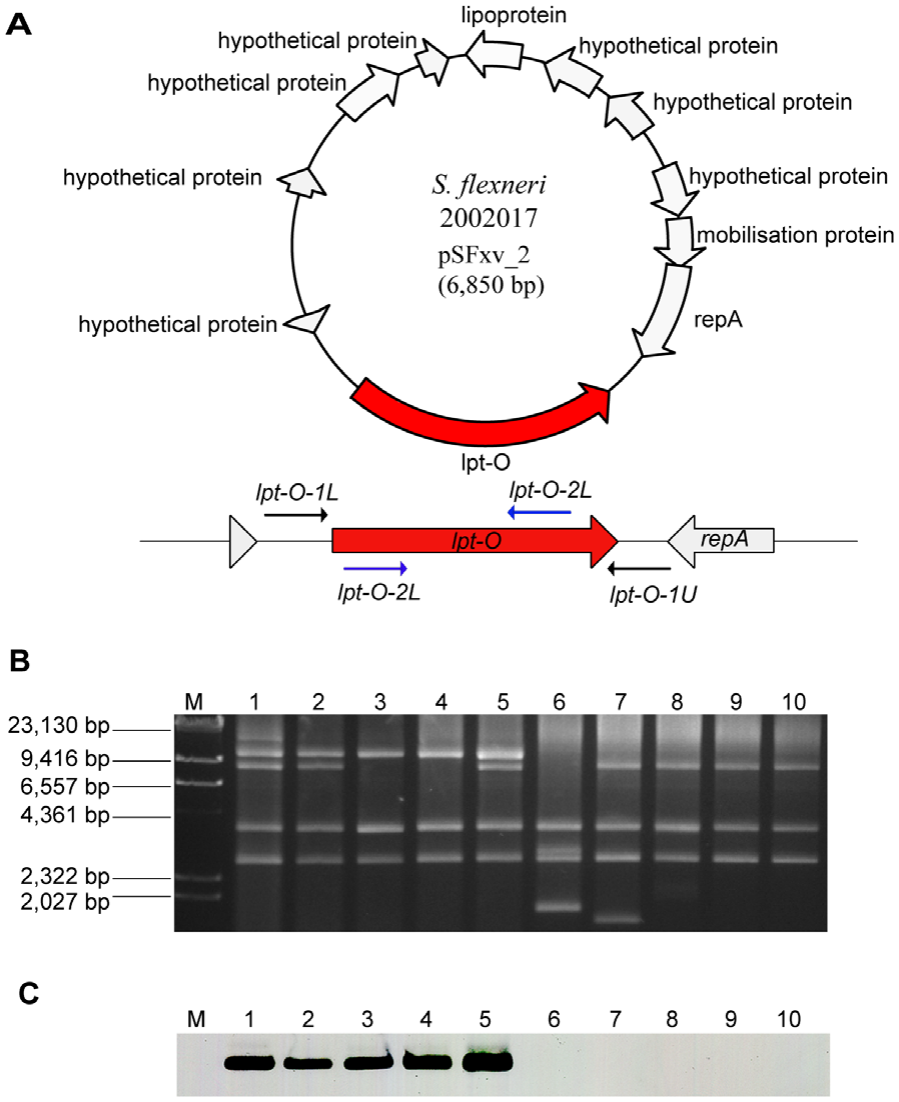
Genomic structure and the presence of plasmid pSFxv_2 in serotype Xv and X strains. **A**, genomic structure of plasmid pSFxv_2. ORFs were shown as thick arrows, and encoding regions were marked according to the genome annotation (accession No. CP001385). The specific *lpt-O* gene was marked in red color, and primers positions were indicated using thin arrows. **B**, plasmid profiles of serotype Xv strains 2002017, 2008129, 2008131, 05AH022 and 05AH027 (lanes 1–5, respectively) and X strains 06HN400, 05BJ002, 03HL001, 03HL020 and 2003055 (lanes 6–10, respectively) strains. Plasmid DNA was separated by electrophoresis with a Chef Mapper system (Bio-Rad) on a 1% SeaKem Gold agarose gel and visualized by EB staining. Plasmids isolated from strain 2002017 (lane 1) were used as control. Lambda DNA cleaved with *HindIII* (TaKaRa, Japan) was used as molecular mass markers. **C**, Southern hybridization detection of the *lpt-O* gene in serotype Xv strains. PCR product amplified from strain 2002017 using primer pair *lpt-O*-2 was prepared as probe.

Chromosomal DNA was isolated from *S. flexneri* strains using DNA extraction Kit (Qiagen, Germany). Oligonucleotide primers *lpt-O*-1U: AAAAATACGCACCGTTCAGA (nt 1271–1290, complementary to pSFxv_2 sequence) and *lpt-O*-1L: AAAAGCACCGTCATCCACTA (nt 3305–3324) were designed for function analysis (Fig. 1A). Primer pair *lpt-O*-2U: ATCTAGTATTGTTGGCGTTA (nt 1721–1740, complementary to pSFxv_2 sequence) and *lpt-O-2*L: CCTTTTCTTGTGTTCTTATC (nt 2799–2818) were used for the *lpt-O* gene detection (Fig. 1A). Oligonucleotide primers were synthesized by Sangon Biotech (Shanghai). PCR amplifications were performed using TaKaRa PCR Amplification Kit (Takara, Japan). Reaction mixtures for each PCR consisted of 1× PCR buffer, 0.2 µM of each primer, 3 µl of template DNA, 2.5 U DNA polymerase and 0.4 mM deoxynucleoside triphosphates in a final reaction volume of 50 µl. Unless otherwise stated, PCR amplification was performed using a standard protocol with the following thermal cycling profile: 94°C for 5 min followed by 30 cycles of 94°C for 30 s, 55°C for 50 s, and 72°C for 2 min, and 72°C for 5 min, on a SensoQuest LabCycler (Germany).

PCR products using primers *lpt-O-*1were purified and cloned into pMD20-T T-A cloning vector (TaKaRa, Japan) to generate the *lpt-O* expression vector pSQZ. The pSQZ was further transformed into ampicillin sensitive strains 51580 (serotype X) and NCTC 9725 (serotype 4a).

### Plasmid Profiling and Southern Hybridization Analyses

A plasmid purification kit (Qiagen, Germany) was used to isolate the plasmids in accordance with the manufacturer’s recommendations. Plasmid DNA was separated by electrophoresis with a Chef Mapper system (Bio-Rad) on a 1% SeaKem Gold agarose gel and visualized by ethidium bromide staining. Plasmids isolated from strain 2002017 were used as the positive control. Lambda DNA cleaved with *HindIII* (TaKaRa, Japan) was used as electrophoresis markers.

The plasmid DNA separated on Gold agarose gel were further transferred onto a nylon membrane (Amersham) using a Vacuum Blotter (Bio-Rad). Southern hybridization was performed using an ECL™ Direct Nucleic Acid Labelling and Detection System (Amersham) as recommended by the manufacturer. DNA product amplified from strain 2002017 using primer pair *lpt-O*-2 was prepared for a biotin-labeling DNA probe.

## Results

### Identification of a Novel O-antigen Modification that gives Rise to the MASF IV-1 Reactivity in Serotype Xv Strains

**Figure 2:**
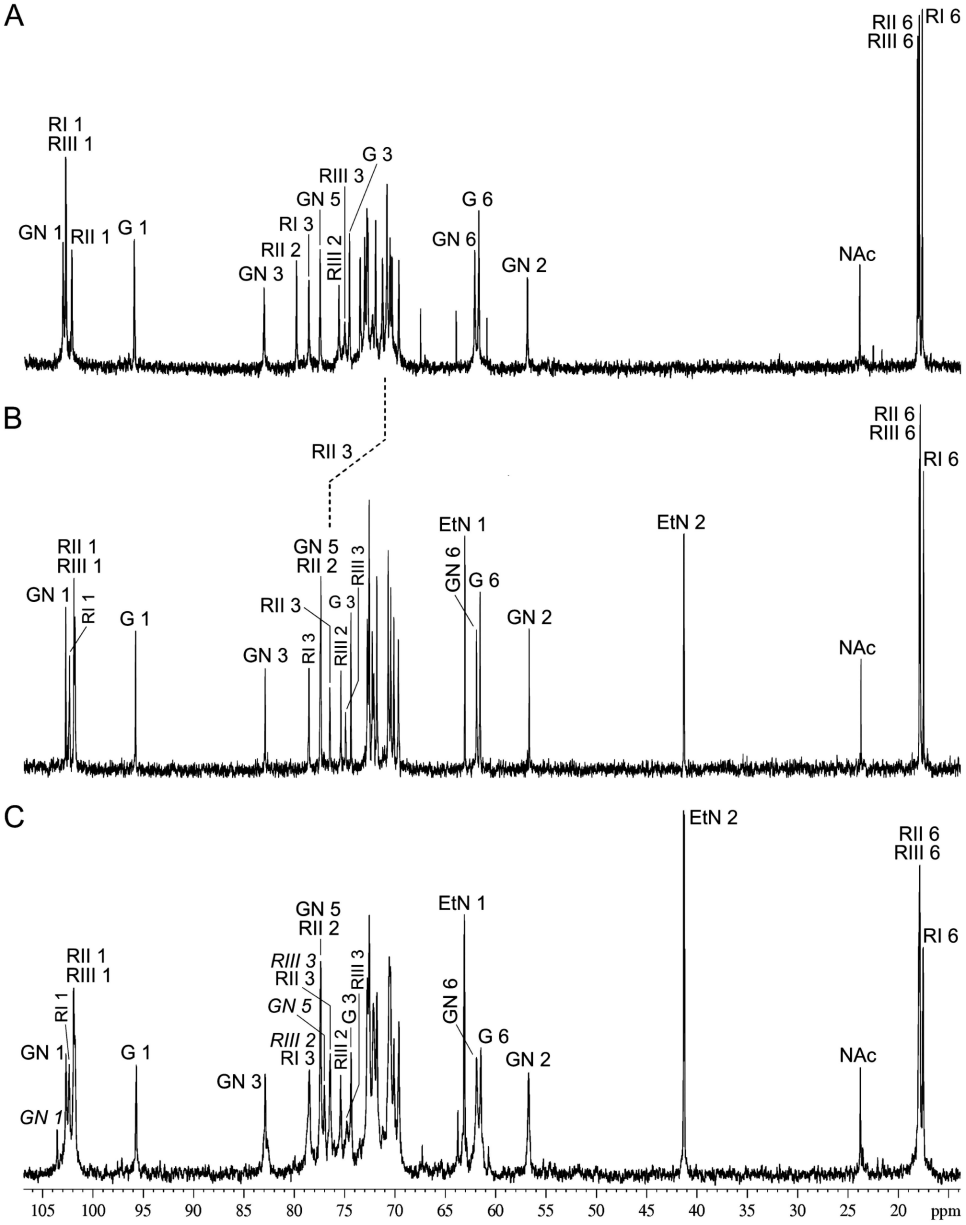
^13^C NMR spectra of the O-polysaccharides from *S. flexneri* serotype X (A), serotype Xv (B) and transformant 51580_Xv (C). Numbers refer to protons in sugar residues denoted as follows: G, Glc; GN, GlcNAc; RI, Rha^I^; RII, Rha^II^; RIII, Rha^III^. In the spectrum of the transformant 51580_Xv O-polysaccharide, the annotations for the signals of the minor bisphosphorylated O-unit are italicized. The most important difference between X and Xv is the presence in the latter of peaks for ethanolamine, which are annotated as EtN 1 and EtN 2. The different positions of the signals for C3 of Rha^II^ (non-phosphorylated in X and phosphorylated in Xv) are shown by a dotted line.

To identify the MASF IV-1 determinant in serotype Xv strains, the isolated O-polysaccharide of serotype Xv strain 2002017 was analyzed by NMR techniques. The ^1^H NMR and ^13^C NMR spectra of the O-polysaccharide demonstrated a non-O-acetylated pentasaccharide O-unit containing one residue each of glucose (Glc) and *N*-acetylglucosamine (GlcNAc) and three residues of Rha (Fig. 2B). However, comparison of the ^1^H NMR and ^13^C NMR spectra of the serotype X O-polysaccharide (Fig. 2A) with those of serotype Xv showed additional signals for a PEtN group at δ_H_ 3.29 and 4.15, δ_C_ 41.4 and 63.2 in the latter (Fig. 2B). There are also other differences in the positions of some signals of Rha^II^ (RII) as well as the intensities of the peaks for Rha^I^ 1 (RI 1), Rha^III^ 1 (RIII 1), and Rha^II^ 1 (RII 1) between X and Xv (Fig. 2), particularly because the peaks for RI 1 and RIII 1 coincided in the spectrum of X, whereas the peaks for RII 1 and RIII 1 coincided in the spectrum of Xv.

**Figure 3:**
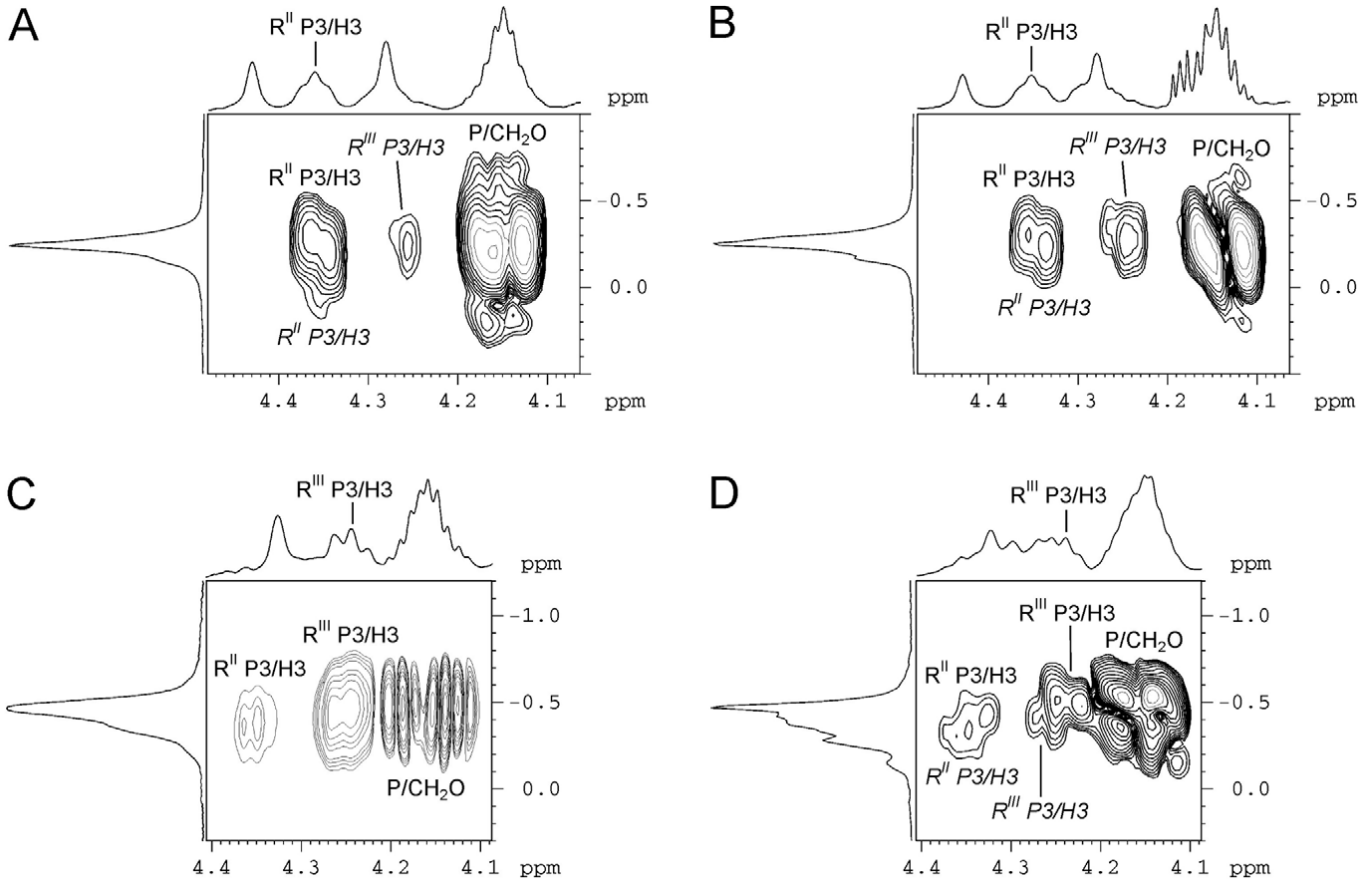
Parts of two-dimensional ^1^H, ^31^P HMBC spectra of the O-polysaccharides from *S. flexneri* serotype Xv (A) and transformant 51580_Xv (B) 4av (C) and transformant NCTC 9725_4av (D). The corresponding parts of the ^1^H NMR and ^31^P NMR spectra are displayed along the horizontal and vertical axes, respectively. RII and RIII indicate Rha^II^ and Rha^III^, respectively. The annotations for the cross-peaks of the minor bisphosphorylated O-unit are italicized.

**Figure 4:**
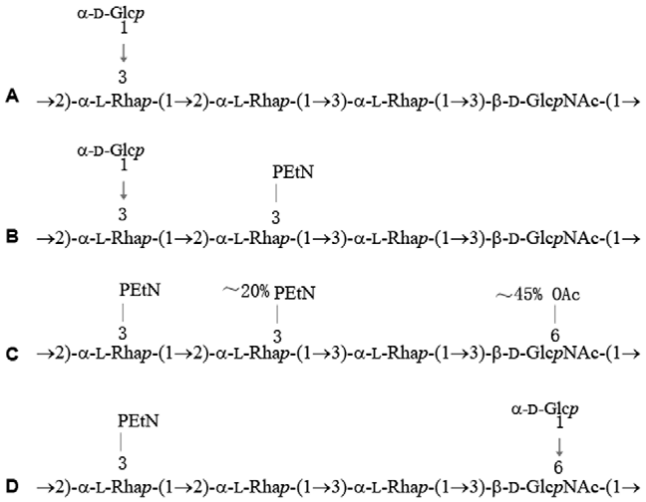
Structures of the O-polysaccharides from *S. flexneri* serotypes X (A) [**21**] **, Xv (B) (this study), Yv (C) (this study), and 4 av (D)** [**29**] **.**

The NMR spectra of the serotype Xv O-polysaccharide were assigned and analyzed using two-dimensional ^1^H,^1^H and ^1^H,^13^C NMR techniques (Table 1) [20]. Significant downfield displacements due to phosphorylation were observed for the signals of H3 and C3 of Rha^II^ to δ_H_ 4.35 and δ_C_ 76.5 as compared with their positions at δ_H_ 3.92 and δ_C_ 71.1, respectively, in the serotype X O-polysaccharide. A two-dimensional ^1^H,^31^P heteronuclear multiple-bond correlation (HMBC) experiment, which correlates by spin coupling phosphorus and protons separated by three bonds, was used to demonstrate independently the site of phosphorylation in the serotype Xv O-polysaccharide. The HMBC spectrum of the O-polysaccharide showed correlations of the phosphate group with CH_2_O protons of the PEtN group at −0.24/4.15 and with H3 of Rha^II^ at −0.24/4.35 (Fig. 3A), respectively. Therefore, PEtN is attached at position 3 of Rha^II^, and the O-polysaccharide of *S. flexneri* serotype Xv has the structure shown in Figure 4B. It differs from the O-polysaccharide of *S. flexneri* serotype X [21] (Fig. 4A) in phosphorylation of Rha^II^ with PEtN only.

**Table 1:** ^1^H and ^13^C NMR chemical shifts (δ, ppm) of *S. flexneri* O-polysaccharides.

Residue	Monosaccharide						EtN/NAc	
	C1	C2	C3	C4	C5	C6	C1	C2
	*H1*	*H2*	*H3*	*H4*	*H5*	*H6(6a,6b)*	*H1*	*H2*
Serotype X								
→2,3)-α-L-Rha*p* ^III^-(1→	102.2	75.6	74.9	72.1	70.7	18.0		
	*5.10*	*4.43*	*3.94*	*3.35*	*3.72*	*1.24*		
→2)-α-L-Rha*p* ^II^-(1→	101.9	79.6	71.1	73.3	70.4	17.9		
	*5.17*	*4.07*	*3.92*	*3.48*	*3.74*	*1.31*		
→3)-α-L-Rha*p* ^I^-(1→	102.2	71.8	78.4	72.9	70.2	17.6		
	*4.85*	*3.84*	*3.78*	*3.53*	*4.02*	*1.23*		
→3)-β-D-Glc*p*NAc-(1→	102.5	56.7	82.8	69.5	77.3	62.0	175.2	23.8
	*4.82*	*3.85*	*3.49*	*3.51*	*3.43*	*3.74,3.90*		2.10
α-D-Glc*p*-(1→	95.7	72.6	74.4	70.7	72.7	61.5		
	*5.18*	*3.72*	*3.83*	*3.49*	*4.04*	*3.78,3.82*		
Serotype Xv								
→2,3)-α-L-Rha*p* ^III^-(1→	101.8	75.4	75.0	72.2	70.7	18.0		
	*5.17*	*4.42*	*3.95*	*3.35*	*3.70*	*1.25*		
→2)-α-L-Rha*p*3PEtN^II^-(1→	101.9	77.5	76.5	72.3	70.5	17.9	63.2	41.4
	*5.15*	*4.27*	*4.35*	*3.60*	*3.80*	*1.33*	*3.29*	*4.15*
→3)-α-L-Rha*p* ^I^-(1→	102.4	71.9	78.6	73.0	70.2	17.6		
	*4.86*	*3.86*	*3.79*	*3.55*	*4.01*	*1.23*		
→3)-β-D-Glc*p*NAc-(1→	102.7	56.8	82.9	69.7	77.4	62.0	175.2	23.8
	*4.82*	*3.84*	*3.48*	*3.49*	*3.42*	*3.73,3.92*		2.10
α-D-Glc*p*-(1→	95.8	72.6	74.4	70.7	72.6	61.6		
	*5.19*	*3.72*	*3.83*	*3.49*	*4.04*	*3.78,3.81*		

The O-antigen of another serotype Xv strain 2003055 was also analyzed and found to have the same structure as that of 2002017, suggesting that the PEtN modification is common among serotype Xv strains and responsible for the MASF IV-1 positive phenotype in serotype Xv.

### A Plasmid Borne Gene (*lpt-O*) is Required for MASF IV-1 Reactivity in Serotype Xv Strains and Conversion of Serotype X to Serotype Xv

Addition of PEtN to the inner core lipopolysaccharide (LPS) or lipooligosaccharide (LOS) has been reported in a number of bacterial species [22], [23], [24], [25], [26], [27] and is carried out by various PEtN transferases. The known genes involved include *lpt3* (NMB2012), *lptA* (NMB1638), *lpt6* (NMA0408) (all *Neisseria meningitides*), *cptA* (STM4118), *pmrC* (ΣΤΜ429) (both *Salmonella* Typhimurium) and *eptB* (ZP_04872785) (*Escherichia coli*) [22], [23], [25]. We used these gene sequences to search the *S. flexneri* 2002017 genome including chromosome and plasmid sequences by tBLASTn and BLASTP for homologous proteins. Seven homologous proteins with 22–99% identity and 39–99% similarity were identified, including 6 from the chromosome and 1 from one of the plasmids, pSFxv_2. The 7 homologs in the 2002017 genome were further compared with other sequenced *S. flexneri* genomes, Sf301, 2457t and 8401, which are all MASF IV-1 negative phenotype strains. All chromosomal homologs were found present in these 3 genomes, whereas the plasmid borne homolog (SFxv_5135) was unique, suggesting that SFxv_5135 may be responsible for the PEtN modification in serotype Xv strains.

To confirm this hypothesis, we first performed PCR assay with 9 more serotype Xv strains (2008129, 2008131, 05AH016, 05AH022, 05AH027, 05AH030, 06HN005, 06HN019 and 2008164 ) and 5 serotype X strains (06HN400, 05BJ002, 03HL001, 03HL020 and 2003055) to determine whether the *lpt-O* gene is carried exclusively by serotype Xv strains. The expected fragment of 1,098 bp can only be obtained from serotype Xv strains, but not from serotype X strains. Sequencing of the PCR products from these 9 serotype Xv strains showed that all were identical to that of 2002017.

Plasmid pSFXv_2 is a double-stranded circular plasmid of 6,850 bp in length. BLAST search revealed no homology with any plasmids or bacterial genomes in the NCBI database, except for 2 regions (4186–5525 and 6058–6847) showing high similarity (96% and 78% identity, respectively) to those of pJHCMW1, a multiresistance plasmid from *Klebsiella pneumoniae* [28]. There are 11 *orfs* coding for a mobilization protein, a replication initiation protein, a lipoprotein and a PEtN transferase discovered in this study as well as 7 proteins of unknown function (Fig. 1A).

To determine whether the *lpt-O* carrying pSFxv_2 plasmid is present in all serotype Xv strains, we analyzed the plasmid profiles of 59 serotype Xv and 40 serotype X strains by electrophoresis with 10 plasmid profiles (serotype Xv strains 2002017, 2008129, 2008131, 05AH022 and 05AH027, and serotype X strains 06HN400, 05BJ002, 03HL001, 03HL020 and 2003055 ) shown in Figure 1B. All strains presented a plasmid profile with 3–7 plasmids, from 2 kb to 10 kb. Note that plasmids larger than 20 kb were not resolvable on this gel and were not included. Compared to serotype X strains, all Xv strains had a 10-kb plasmid band (Fig. 1B). Southern hybridization using biotin-labeled *lpt-O* as a probe confirmed that the 10-kb plasmid band corresponds to the *lpt-O*-carrying pSFxv_2 of 6.85 kb as only Xv strains showed a positive signal (Fig. 1C). The 10-kb plasmid band of 2002017 was extracted from gel and used as template for overlapping PCR covering the circular plasmid. Results indicated that the plasmid sequence is identical to that of pSFxv_2 (unpublished data). The observation that the plasmid appeared larger in size on the gel than its actual size of 6.85 kb may be due to the migration difference of covalently closed circular plasmid DNA and the molecular marker of linear DNA. The data obtained suggest that all serotype Xv strains carry a pSFxv_2-like plasmid, which mediates the addition of PEtN to the O-antigen giving rise to the MASF IV-1 positive phenotype.

To elucidate the function of the *lpt-O* gene, we then cloned the entire *lpt-O* gene of 1,521 bp, together with 533 bp sequences up and downstream to cover its promoter and terminator sequences, from strain 2002017 into plasmid pMD20-T (pSQZ) and transformed it into serotype X strain 51580 (amp^s^). Slide agglutination assay indicated that the host strain was converted to serotype Xv, with the transformant (51580_Xv) serologically reacting with both MASF IV-1 and MASF 7,8 monoclonal antibodies (Table 2). Similar to that of serotype Xv strains, the transformant also reacted with monovalent antisera IV of Denka Seiken (Table 2). The serological features of 51580_Xv were identical to that of the control strain 2002017 (Table 2).

**Table 2:** Serological identification of transformants using the monoclonal antibodies of MASF scheme and monovalent antisera of Seiken by slide agglutination.

Strains	Serotype	Reaction with monoclonal antibody of MASF											Reaction with monovalent antisera of Seiken										
		Typing antibodies					Grouping antibodies				1C			Typing antisera							Grouping antisera		
		I	II	IV-2	V	VI	Y-5	6	7,8	IV-1				I		II	III	IV	V	VI	3,4	6	7,8
51580	X	−	−	−	−	−	−	−	+	−	−		−		−		−	−	−	−	−	−	+
51580_Xv	Xv	−	−	−	−	−	−	−	+	+	−		−		−		−	+	−	−	−	−	+
2002017	Xv	−	−	−	−	−	−	−	+	+	−		−		−		−	+	−	−	−	−	+
NCTC 9725	4a	−	−	+	−	−	+	−	−	-	−		−		−		−	+	−	−	+	−	−
NCTC 9725_4av	4av	−	−	+	−	−	+	−	−	+	−		−		−		−	+	−	−	+	−	−
G1668	4av	−	−	+	−	−	+	−	−	+	−		−		−		−	+	−	−	+	−	−

A previous study has identified that serotype 4a strain G1668 contains a PEtN residue on Rha^III^ at position 3 [29]. We confirmed serologically that G1668 is MASF IV-1 positive and thus is a serotype 4a variant hereafter called 4av. To examine whether the same gene can convert serotype 4a into serotype 4av, a serotype 4a strain NCTC 9725 (amp^s^) was transformed with pSQZ. The transformant (NCTC 9725_4av) showed the acquired MASF IV-1 positive phenotype with the same serological feature as that of control serotype 4av strain G1668 (Table 2).

Motif analysis revealed that the *lpt-O* encoding protein contains a sulfatase domain on its carboxyl terminal and thus belongs to the sultafase superfamily. At protein level, Lpt-O shows significant similarity with a sulfatase protein (71% identity and 85% similarity) of Enterobacteriaceae bacterium 254FAA (ZP_07953373) and is moderately homologous to sulfatase proteins (31–38% identity, and 51–56% similarity) in *E. coli*, *Erwinia billingiae*, *Pantoea vagans*, *Stenotrophomonas maltophilia*, *Eubacterium cellulosolvens* and *Afipia s*p. The sulfatase domain is also present in the other known PEtN transferases (Lpt3, Lpt6, LptA, CptA, EptC, EptB, PmrC, Hp0022) [22], [23], [25], [26], [27], [30], [31], [32] and is likely to be involved in catalyzing the transfer of PEtN moiety to a sugar residue.

### Gain of the *lpt-O* Gene and MASF IV-1 Reactivity was Concomitant with Gain of the O-antigen Modification with PEtN

To confirm that the gain of the *lpt-O* gene and MASF IV-1 reactivity by serotype X strains correlates with the gain of PEtN, the O-polysaccharide of the transformant 51580_Xv was analyzed by NMR spectroscopy. The ^1^H NMR and ^13^C NMR (Fig. 2C) spectra of the 51580_Xv O-polysaccharide showed a structural heterogeneity. Analysis of the spectra using two-dimensional NMR techniques revealed the occurrence of two types of repeats in the ratio of ∼1.5∶1: the major O-unit typical of the Xv O-polysaccharide (Fig. 4B) and a minor bisphosphorylated non-glucosylated O-unit. Particularly, in addition to the major signals for the phosphorylated Rha^II^ residue (δ_H-3_ 4.35 and δ_C-3_ 76.5), there were signals for the phosphorylated Rha^III^ residue (δ_H-3_ 4.25 and δ_C-3_ 76.2). Accordingly, in the ^1^H,^31^P HMBC spectrum (Fig. 3B), there were one major P/Rha^II^ cross-peak at δ −0.24/4.35 for the monophosphorylated O-unit and two minor P/Rha^II^ and P/Rha^III^ cross-peaks at δ −0.16/4.35 and −0.24/4.25, respectively, for the bisphosphorylated O-unit. A more careful analysis of the NMR spectra, including the ^1^H,^31^P HMBC spectrum, indicated the presence of the bisphosphorylated O-units in the wild-type Xv O-polysaccharide too but their content is significantly lower (∼15% of the total O-units versus ∼40% in the transformant 51580_Xv O-polysaccharide) (Fig. 3A). Therefore, in serotype Xv strains phosphorylation interferes with glycosylation on Rha^III^ giving rise to the O-units in which the lateral Glc residue is replaced with the second PEtN group. The bisphosphorylated O-units of this type are characteristic for some strains of serotype Y that may be called Yv (see below) (Fig. 4C) (authors’ unpublished data), which seems to be the same as reported serotype 4 s [33].

NMR spectroscopic analysis showed that the major O-unit of the NCTC 9725_4 av transformant (∼50% of the total O-units) has the same structure as that of wild-type 4 av strains reported earlier [29] (Fig. 4D). Minor O-unit variants (each of ∼25%) lack lateral Glc and have either one PEtN group on Rha^II^ or two PEtN groups on both Rha^II^ and Rha^III^ (for the ^1^H,^31^P HMBC spectrum see Fig. 3D). Again, a more careful analysis of the wild-type 4 av polysaccharide revealed the presence of minor proportion (∼10%) of the O-units with PEtN groups on Rha^II^ (for the ^1^H,^31^P HMBC spectrum of the O-polysaccharide from strain G1668 see Fig. 3C). Therefore, the *lpt-O* gene is required and sufficient for the addition of PEtN residue to the O-antigen in serotype 4 av strains too. These data also suggest that Lpt-O can mediate the attachment of PEtN to either Rha^II^ or Rha^III^.

### The Functional *lpt-O* Gene is Carried Only by MASF IV-1 Positive Phenotype Strains

To determine whether the *lpt-O* gene is more widely present in *S. flexneri*, another expanded PCR screening of 310 strains of 15 serotypes (Table 3) was performed using primer pair *lpt-O*-2. These strains were randomly selected from our collection, and their serotype characteristics have been determined earlier. A strict correlation was observed between the presence of functional *lpt-O* gene and MASF IV-1 positive reactivity. As expected, PCR products were found in all 59 serotype Xv strains tested. The expected PCR product (1,098 bp) was also obtained from 19 serotype Yv, 3 serotype 4 av (2002091, NCTC 8296 and G1668), and surprisingly, 4 serotype X (HN059, HN060, HN066 and HN378) and one serotype 4b (NCTC 9726) strains. Although the *lpt-O* gene can be amplified from these 5 strains, they all did not react with monoclonal antibody MASF IV-1. Plasmid profile analysis indicated that the pSFxv_2- like plasmid is present in these 5 strains. Sequencing of the *lpt-O* gene from these 5 strains revealed that all had a single base deletion at the position of 295, which resulted in a stop codon at amino acid 99, and abolishes the function of the gene without the presumed sulfatase domain (from aa165 to aa465) of Lpt-O. We cloned the defective gene and showed that it cannot convert serotype X strain 51580 to serotype Xv, confirming that the single base deletion inactivated the gene.

**Table 3:** Distribution of the *lpt-O* gene in *S. flexneri* strains detected by PCR.

Serotypes	Number of strains tested	Number of serological reactive strains with^1^		*lpt-O* gene PCR results^2^
		MASF IV-1	IV	
1a	25	–	–	–
1b	17	–	–	–
1c	2	–	–	–
1d	5	–	–	–
2a	24	–	–	–
2b	27	–	–	–
3a	8	–	–	–
3b	2	–	–	–
4a	3	–	3	–
4av^3^	3	3	3	+(3)
4b	5	–	–	−/+(1)
5a	4	–	–	–
X	70	–	–	−/+(4)
Xv	59	59	59	+(59)
Y	15	–	–	–
Yv^4^	19	19	19	+(19)
6	23	–	–	–

1 Reactivity with monoclonal antibody MASF IV-1 (Reagensia AB) and monovalent antisera IV (Denka Seiken). Numbers of *S. flexneri* strains with MASF IV-1 positive or IV+ phenotype were listed.

2 + and −/+ indicate the presence of the functional and inactivated *lpt-O* gene, respectively. Number of strains carrying the *lpt-O* gene is indicated in parentheses.

3 Serotype 4 av strains (2002091, NCTC 8296 and G1668) were serologically different from typical serotype 4a strains in the MASF IV-1 reactivity.

4 Serotype Yv strains were serologically different from typical serotype Y strains in the MASF IV-1 and monovalent antisera IV reactivity.

The *lpt-O* positive serotype Yv strains are serologically different from typical serotype Y with additional MASF IV-1 and monovalent antisera IV reactivity phenotype. Analysis of the O-antigen structure of a serotype Yv strain, HN006, demonstrated a PEtN modification at position 3 of both Rha^II^ and Rha^III^ (Fig. 4C) (details of structural studies on the Yv O-antigen will be reported elsewhere). The 3 *lpt-O* positive genotype 4 av strains differ from typical serotype 4a strains only in the MASF IV-1 positive phenotype (Table 2). The *lpt-O* gene in these 3 serotype 4 av strains were sequenced and compared to that of 2002017. The *lpt-O* genes among the 3 serotype 4 av strains are identical, and they are highly homologous to that of 2002017 (99.3%), with 11 base changes (243, A→G; 310, G→A; 379, G→A; 687, T→G; 691, T→C; 728, C→T; 772, C→T; 836, C→A; 1144, G→A; 1449, G→A; 1481, T→C), which resulted in the substitution of 7 amino acids of Lpt-O (104, V→I; 127, E→K; 231, Y→H; 243, A→V; 279, A→D; 382, V→I; 494, I→T). It is not certain if these changes would have an effect on the specificity of PEtN addition to Rha. However, the observation that cloned *lpt-O* from serotype Xv strain 2002017 can mediate the phosphorylation modification on both Rha^II^ and Rha^III^ of transformant NCTC 9725_4 av and 036_Yv (unpublished data) indicates the common function of the *lpt-O* homologues.

## Discussion

The O-antigen structure and its diversity of *S. flexneri* are well studied. Until recently, glucosyl and *O*-acetyl groups have been the only two known residues attached to *S. flexneri* O-antigen backbone giving rise to various serotypes. In 2009, Perepelov et al [29] had reported the ethanolamine phosphate (EtnP) attachment at position 3 of Rha^III^ of the O-antigen in two serotype 4a strains G1668 and 1359. However, the mechanism of PEtN modification has not been elucidated. In this study, the novel O-antigen modification, πηοσπηορψλατιον ωιτη ΠΕτΝ, was identified in serotype Xv and some other serotypes. PEtN modifications have been reported in other bacteria, including *N.meningitidis* [27], *Haemophilus influenzae* [34], *S.* typhimurium [23], *E. coli* [31], *Pasteurella multocida* [35] and *Campylobacter jejuni* [32]. However, the PEtN modification in these cases occurs in the LPS or LOS core and does not involve in serotype conversion except in *P. multocida* [35]. Our study revealed other serotypes of *S. flexneri* modified by PEtN through PCR screening, and potentially there are many variants of the currently known serotypes due to PEtN modification. As reported previously [2], serotype Xv emerged as a new serotype and rapidly became the predominant serotype in China to cause a large number of shigellosis cases, the other variants also have the potential to flourish and change the dynamics of *S. flexneri* epidemics. The elucidation of this new O-antigen modification mechanism provides avenue for further study of *S. flexneri* serotype diversity and epidemiology.

The presence and location of non-carbohydrate substituents in LPS, such as PEtN, are critical for the immune recognition of LPS by antibodies in some bacterial species [27], [35]. The PEtN-3 constituent on the β-chain heptose (HepII) of the inner core LPS of *N. meningitides* is the target epitope of protective antibody mAb B5+ [27]. In *P. multocida*, the serological classification of strains into serotypes 2 and 5 is dependent on the presence or absence of PEtN on Hep II of LPS [35] **.** In this study, the MASF IV-1 positive reactivity was found to be dependent on the PEtN modification of the O-antigen, which confers the specific MASF IV-1 or E1037 antigen on the host. Since human immune response to *S. flexneri* infection is serotype specific with protection against further infection by the same serotype only [36], the presence of a new surface epitope in *S. flexneri* would expect to confer the bacterium an adaptive advantage to evade human immune killing. This may account for the emergence of serotype Xv in China, which appeared in one province initially and expand to most provinces of China during a short period of time, surpassing serotype 2a as the predominant serotype [2]. Apart from the multidrug resistance of the new serotype [2], the immune evasion offered by the new PEtN modification may also play an important role in the spread of the pathogen.

In this study, the *lpt-O* gene required for the PEtN addition was also identified. Its causal role is supported by the following evidences: 1) the protein encoded by the *lpt-O* gene is homologous to other PEtN transferases; 2) the cloned *lpt-O* gene can transform serotype X to serotype Xv and serotype 4a to serotype 4 av; 3) the PEtN modification correlates strictly with the presence of the functional *lpt-O* gene; 4) naturally occurring mutation (one base deletion) abolishes the function of the *lpt-O* gene without the presumed sulfatase domain and lost the reactivity with MASF IV-1 antibody. Future studies are necessary to characterize the biochemical process of the PEtN modification.

The known O-antigen modifications through glucosylation or O-acetylation in *S. flexneri* are mediated by serotype-converting bacteriophages or prophages with 7 identified up to date [3], [6], [8], [9], . In contrast, our study demonstrated a plasmid-encoded serotype conversion mechanism, which has not been reported in *S. flexneri* earlier. This finding changed the landscape of *S. flexneri* serotype diversity and serotype conversion. As an easily transferable factor, the *lpt-O* carrying plasmid can spread among different stains and mediate the serotype conversion. Serotype Xv strains are clearly originated from serotype X strains by gaining a *lpt-O* carrying plasmid since previous PFGE analysis showed that serotype Xv strains were grouped together with serotype X, and many serotype Xv strains shared the same PFGE profiles as serotype X strains [2].

In conclusion, the chemistry and genetics of a new O-antigen modification in *S. flexneri* serotype Xv have been elucidated. Attachment of a PEtN group to Rha residues gives rise to the MASF IV-1 positive phenotype in this and several other serotypes. The *lpt-O* gene, required for PEtN addition is carried by a pSFxv_2 like plasmid in all MASF IV-1 positive phenotype strains. This novel mechanism of modification has profound implications in *S. flexneri* epidemiology as it may offer a significant advantage to the bacterium which is demonstrated by the prevalence of serotype Xv in China. Additionally, these findings, together with published data [2], [15], [17], [33], show a necessity of further extension of classification scheme for *S. flexneri* by adding MASF IV-1 positive strains as new subtypes of serotypes 4, X and Y.
